# Perspectives for immunotherapy of EBV‐associated GLELC: A relatively “hot” tumor microenvironment

**DOI:** 10.1002/cam4.6555

**Published:** 2023-09-21

**Authors:** Yanna Lei, Peng Cao, Xiufeng Zheng, Jing Wei, Mo Cheng, Ming Liu

**Affiliations:** ^1^ Department of Gastric Cancer Center, West China Hospital Sichuan University Chengdu Sichuan China; ^2^ Department of Abdominal Oncology, West China Hospital Sichuan University Chengdu Sichuan China; ^3^ Department of Colorectal Cancer Center, West China Hospital Sichuan University Chengdu Sichuan China

**Keywords:** angiogenesis, Epstein–Barr virus (EBV)‐associated gastric lymphoepithelioma‐like carcinoma, gastric adenocarcinoma, tumor microenvironment, tumor‐infiltrating lymphocytes

## Abstract

**Background:**

Epstein–Barr virus (EBV)‐associated gastric lymphoepithelioma‐like carcinoma (EBVaGLELC) represents a small number of gastric cancer (GC), and research on tumor microenvironment (TME) and treatment strategy are still lacking.

**Aims:**

Here, we aim to elucidate the immune features of this rare disease and further help to develop more effective treatment options.

**Materials & Methods:**

A retrospective analysis was conducted between 2019 to 2022 in West China Hospital to reveal the immunological characteristics of EBV‐positive GLELC. The difference of immune cell subset and tumor vascular structure between gastric denocarcinoma (GAC) and EBVaGLELC will be pointed out.

**Discussion:**

13 patients with GELEC and 8 patients with GAC were retrospectively studied. The heterogeneity of the immune cell profile was then confirmed through multiplexed immunofluorescence staining (mIF), which revealed a higher proportion of CD3^+^ T cells, CD8^+^ T cells, and Treg cells in the EBV‐associated GLELC group. Such a distinct TME may provide therapeutic advantages, and patients with this rare subtype of GC could be good candidates for immune checkpoint inhibitors (ICIs). Angiogenesis in EBV‐positive GLELC may be less intense than that in gastric adenocarcinoma (GAC), a feature that might decrease their susceptibility to antiangiogenic therapy. Furthermore, we reported a 52‐year‐old male with advanced EBV‐positive GLELC who showed a favorable response to the combined therapy with . A repeat evaluation showed sustained partial response (PR), and the progression‐free survival (PFS) was more than 34 months until now.

**Conclusion:**

Compared with GAC, EBVaGLELC revealed higher T cell infiltration and less intense of angiogenesis. It displays relatively “hot” TME that may provide the rationality to treat with immunotherapy in EBV‐related GLELC.

## Graphical abstract

Compared with gastric adenocarcinoma, EBV‐positive GLELC has distinct TME characteristics. A “hot” TME may provide therapeutic advantages for those patients. Furthermore, our case provides an underlying therapeutic option for patients with EBV‐associated GLELC. This study supplements the limited literature, which could also contribute to the clinic's treatment decision‐making, especially for advanced patients.

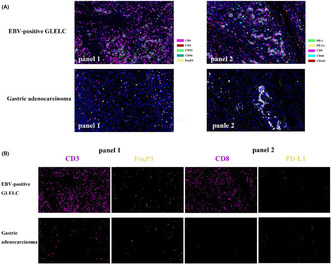

## 1 BACKGROUND

Gastric lymphoepithelioma‐like carcinoma (GLELC) is a rare subtype of gastric cancer (GC) that constitutes only 1%–4% of all GCs.^1^, ^2^, ^3^ More than 80% of GLELC is closely associated with Epstein–Barr virus (EBV) infection.^4^ EBV is a human cancer‐associated virus that can lead to the transformation of epithelial cells into permanently proliferating cells and thus cause the formation of epithelial cell derived malignancies such as nasopharyngeal carcinoma (NPC) and GC.^5^ A new molecular classification of GC was proposed in 2014, and EBV‐associated GC is one of four major types.^6^ It was reported that EBV‐associated GC has different biological characteristics from other types of GC. A lower tumor‐node‐metastasis (TNM) stage, higher level of programmed death ligand‐1 (PD‐L1) expression, and survival advantage were found in EBV‐positive patients.^3^, ^7^, ^8^

Most GC patients are not considered for surgery at the time of diagnosis. For advanced‐stage GC, chemotherapy‐based comprehensive treatment is the main treatment method.^9^ However, for EBV‐associated GLELC (EBVaGLELC), which is commonly reported in case reports and small series, there is no standard therapy, and practitioners lack a reference point for determining the treatment strategy. Immunotherapy, especially immune checkpoint inhibitors (ICIs), heralded a new era of advanced cancers, including gastric adenocarcinoma (GAC),^10^, ^11^, ^12^, ^13^, ^14^ but the efficacy in EBVaGLELC has not been well characterized. The tumor microenvironment (TME), a novel hallmark of cancer, is closely correlated with cancer formation and progression. The TME provides a sustained favorable environment for tumor growth and consists of endothelial cells, fibroblasts, pericytes, adipocytes, immune cells, cancer stem cells, and vasculature.^15^ Increasing evidence indicates that TME components influence the patient response to tumor treatment, such as immunotherapy.^16^ Understanding the crosstalk between cancer cells and immune cells in the TME is a key issue in cancer immunity.^17^, ^18^, ^19^ However, due to the rarity of this disease, the immune cell‐related characteristics and tumor vascular structure remain largely unexplored.

Of note, the reported rate of EBVaGLELC in patients of Chinese descent has increased in recent years. Thus, a systematic study is required to elucidate the immune features of this rare disease and further help to develop more effective treatment options. In this study, we aimed to comprehensively characterize the clinical characteristics, immune cells, tumor vascular structure, and expression of PD‐L1 in the TME of EBVaGLELC. Furthermore, we report on an EBVaGLELC patient with a PFS of more than 34 months to date who exhibited a marked response to combined therapy with immune checkpoint blockade.

## 2 PATIENTS AND METHODS

### 2.1 Patients, samples, and data collection

Patients with EBVaGLELC were retrospectively studied in West China Hospital between January 2019 and 2022. The inclusion criteria were as follows: (1) a histologically confirmed diagnosis of EBV‐positive GLELC in West China Hospital; (2) age ≥18 years; (3) EBV status was identified using the polymerase chain reaction (PCR) technique to detect EBERs in tissue samples; (4) no prior radiotherapy, chemotherapy, or any other targeted therapies for the current gastric cancer; and (5) patient blocks were sufficient to perform mIF analysis. Patients with incomplete data were eliminated from this study, and finally, a total of 13 cases of gastric primary LELC were included. To define the distinction of the TME between GELEC and GAC more thoroughly, patients with GAC were also included in our study. Randomly selected GAC patients (*N* = 8) were eligible if they were aged ≥18 years, histologically confirmed diagnosis and blocks were sufficient to perform mIF analysis. All samples were gastrectomy or gastric biopsy samples and were obtained prior to treatment at the Department of Pathology, West China Hospital between 2019 and 2022. The clinical features of these individuals, including baseline characteristics and tumor‐related information, were retrieved from our hospital's electronic medical record system.

### 2.2 Ethical approval

The present study was approved and authorized by the institutional review board of West China Hospital (2022–1404).

### 2.3 Multiplexed immunofluorescence staining

The blocks of the above 21 patients were analyzed through mIF. All steps were performed on a Leica Bond Rx autostainer (Leica Microsystems), and then, images were acquired on an Akoya Vectra Polaris. The HALO image analysis platform (Indica Labs) was used for image analysis. This study includes antibodies against: CD163(ab182422), CD20(L26 IR604), CD3(A0452 IR503), CD4(ab133616), FoxP3(ab20034), CD56(ab75813), CD68(ab213363), CD8(ab178089), PD‐1(D4W2J), and PD‐L1(E1L3N). Immune cells were analyzed, including CD3^+^ T cells, helper T cells (CD3^+^, CD4^+^), CD8^+^ T cells, regulatory T (Treg) cells (CD3^+^, CD4^+^, FoxP3^+^), PD‐1^+^ effector T lymphocytes (PD‐1^+^, CD8^+^), M1‐like tumor‐associated macrophages (TAMs) (CD68^+^, CD163^−^), M2 TAMs (CD68^+^, CD163^+^), PD‐L1^+^ macrophages (PD‐L1^+^, CD68^+^), NK cells (CD56 bright and CD56 dim), and B lymphocytes (CD20). Cancer‐associated fibroblasts (CAFs) are associated with tumor growth, invasion, and metastasis and are characterized by high α‐SMA expression.^20^, ^21^ CD31 staining was used to evaluate microvessel density (MVD).^22^ The coexpression of CD31 and α‐SMA was examined using immunofluorescence double staining. E1L3N was used to evaluate PD‐L1 expression status, and a combined positive score (CPS) was calculated. CPS could assess PD‐L1 expression on both tumor cells and immune cells, and a CPS score of ≥1 was interpreted as positive PD‐L1 expression.^23^

### 2.4 Statistical analysis

The counts and percentages of immune cells were obtained, and statistical analyses were performed using R v.4.0.3 (https://www.r‐project.org) and GraphPad Prism 8 software. Unless otherwise indicated, data are expressed as the means ± SDs or means ± SEMs. A *p* < 0.05 was considered statistically significant.

## 3 RESULTS

### 3.1 Patient characteristics

The mean age of patients (*n* = 13) who developed EBV‐associated GLELC was 59.69 ± 7.70 years, with 84.6% males (Table 1). Among the 13 patients, 5 (38.5%) had a large tumor size, with a mean size of approximately 5.04 ± 3.38 cm in diameter. A total of 76.9% were in the proximal stomach, and approximately half were G3 tumors. According to the eighth edition AJCC staging system, 9 (69.2%) cases were in the T3–T4 stage, and 61.2% were in the N0‐N1 stage. A total of 92.3% of patients did not have any distant metastasis and received surgery. The patients in the GAC group had a mean age of 61.88 years, and 87.7% of the patients were male. Compared to EBVaGLELC, GAC had a relatively small tumor size (*p* = 1.00), a high percentage of advanced T stage (*p* = 0.699), less positive node metastasis *(p* = 0.377), and well cell differentiation (*p* = 0.25). EBV infection was not found in patients with GAC, and most were MMR‐proficient patients. The HER‐2 status in most patients was negative. Differences between the two types of GC are summarized in Table 1.

**TABLE 1:** Baseline characteristics of patients in the study (*n* = 13).

Variable	EBVaGLELC (*N* = 13)	GAC (*N* = 8)	*p*‐value (%)
Age			
<65	9 (69.2)	4 (50.0)	0.676
≥ 65	4 (30.8)	4 (50.0)	
Mean(SD)	59.69 years (7.70)	61.88 years (12.67)	
Sex			
Female	2 (15.4)	1 (12.5)	1.00
Male	11 (84.6)	7 (87.5)	
Tumor size			
≥5	5 (38.5)	3 (37.5)	1.00
<5	8 (61.5)	5 (62.5)	
Mean(SD)	5.04 (3.38)	4.20 (1.73)	
Tumor site			
Proximal stomach	10 (76.9)	1 (12.5)	0.015
Distal stomach	3 (23.1)	7 (87.5)	
Cell differentiation			
G1	0 (0.0)	0 (0.0)	0.25
G2	0 (0.0)	3 (37.5)	
G3	9 (69.2)	5 (62.5)	
Unknown	4 (30.8)	0 (0.0)	
Surgery			
No	1 (7.7)	1 (12.5)	1.00
Yes	12 (92.3)	7 (87.5)	
T stage			
T1‐2	4 (30.8)	1 (12.5)	0.669
T3‐4	9 (69.2)	7 (87.5)	
N stage			
N0	4 (30.8)	5 (62.5)	0.377
N1	4 (30.8)	1 (12.5)	
N2	2 (15.4)	0 (0.0)	
N3	3 (23.1)	2 (25.0)	
M stage			
M0	12 (92.3)	7 (87.5)	1.00
M1	1 (7.7)	1 (12.5)	
HER‐2 status			
Positive	2 (15.4)	0 (0.0)	0.167
Negative	10 (76.9)	8 (100.0)	
Unknown	1 (7.7)	0 (0.0)	
EBER status			
Negative	0 (0.0)	8 (100.0)	<0.001
Positive	13 (100.0)	0 (0.0)	
MMR			
dMRR	1 (7.7)	1 (12.5)	1.00
pMMR	12 (92.3)	7 (87.5)	

### 3.2 PD‐1 and PD‐L1 expression levels in EBV‐associated GLELC

PD‐L1 serves as a ligand of programmed cell death protein 1 (PD‐1), which is expressed on tumor cells or stromal immune cells. PD‐L1 can inhibit the activation of cytotoxic T cells by combining with PD‐1 and contribute to evading immune surveillance. Here, we assessed PD‐L1 expression in tumor cells and immune cells through immunohistochemistry (IHC) analysis for all samples. No significant difference was observed between the PD‐L1 expression levels in the tumor cells. A substantial proportion of patients did not express high levels of PD‐L1 in both groups either group. PD‐1 is mainly expressed in T cells. The expression of PD‐1 was higher in the GAC group, but there was a significant difference between those two groups (*p* = 0.04).

**FIGURE 1:**
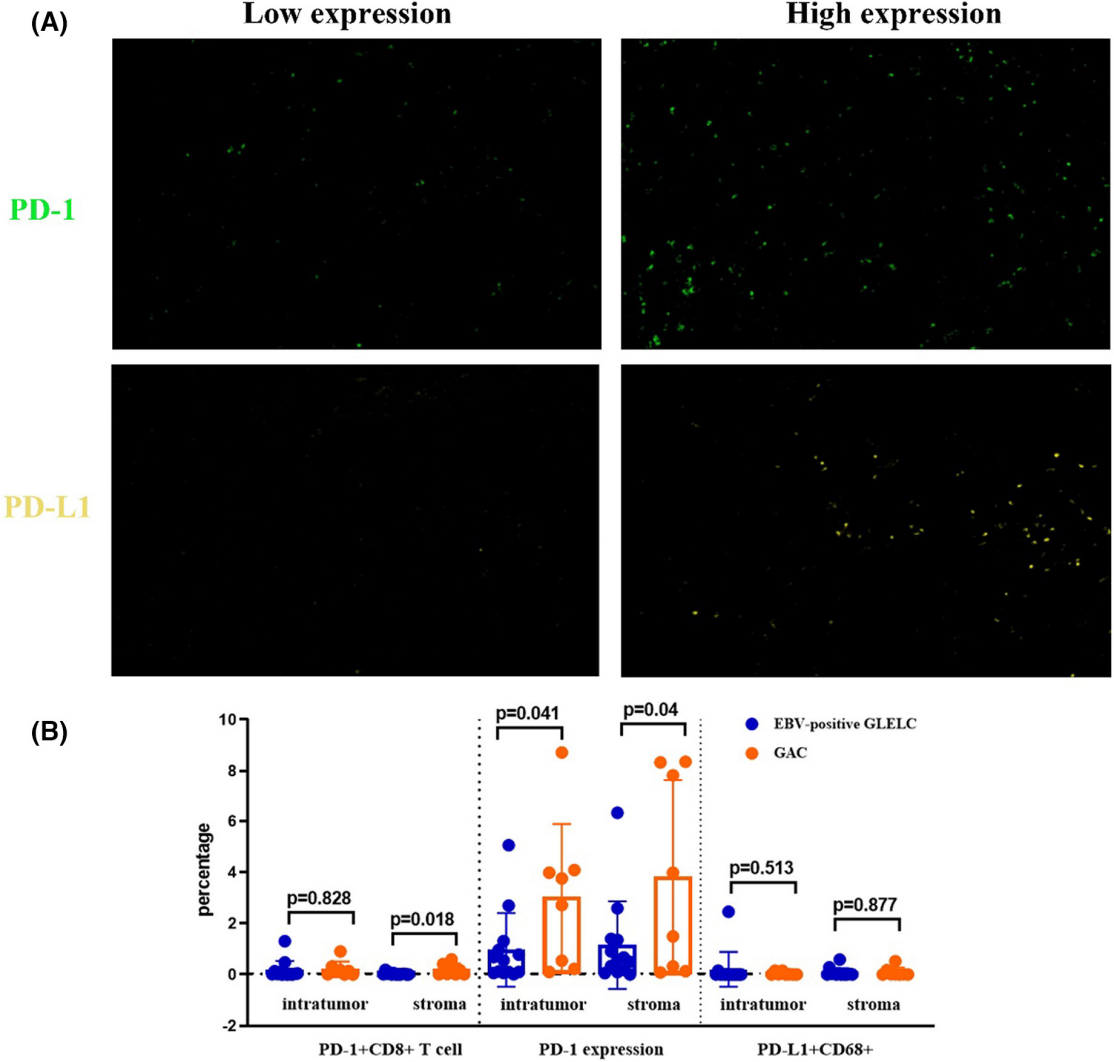
(A) Representative high expression and representative low expression of PD‐1 and PD‐L1. ×200 magnification. (B) The expression status of PD‐1 + CD8+ T cells and PD‐L1 + CD68+ cells in the tumor region and stroma. Each dot represents one sample, and the data are presented as the mean ± SEM. A *p* value <0.05 was considered to be statistically significant.

Immune cell PD‐L1 colocalization with macrophages (PD‐L1 + CD68+) was also measured in our study. Consequently, no statistically significant difference was found (*p* = 0.513). The percentages in the tumor region were 0.2% and 0.04%, respectively. The levels of expression (low or high) of PD1 and PD‐L1 are shown in Figure 1.

### 3.3 Features of the tumor microenvironment in patients with EBV‐associated GLELC

**FIGURE 2:**
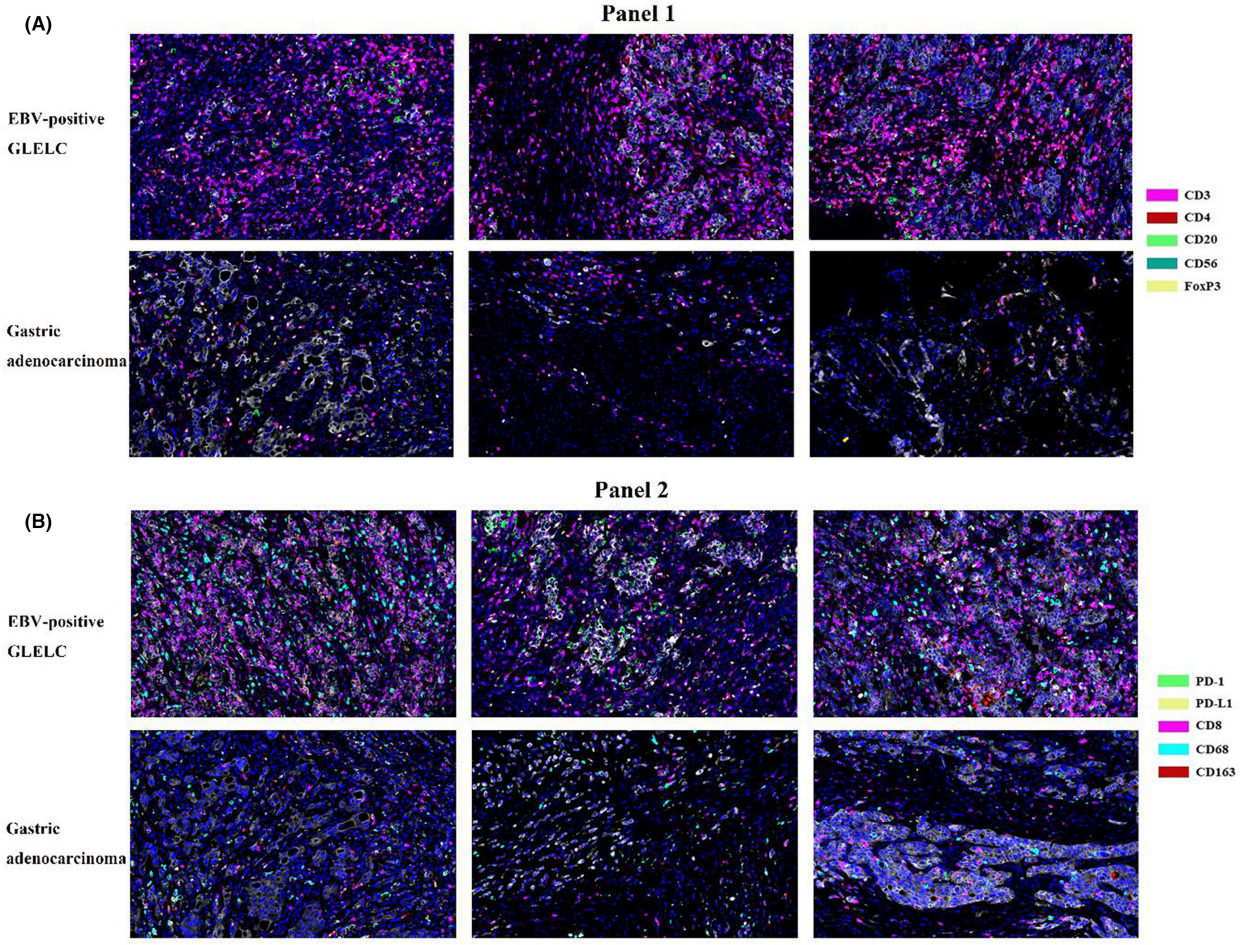
(A) Representation of six examples of CD3, CD4, FOXP3, CD20, and CD56 (Panel 1). (B) CD8, PD‐1, PD‐L1, CD68, and CD163 (Panel 2) expression levels in EBV‐associated GLELC and gastric adenocarcinoma. ×200 magnification.

**FIGURE 3:**
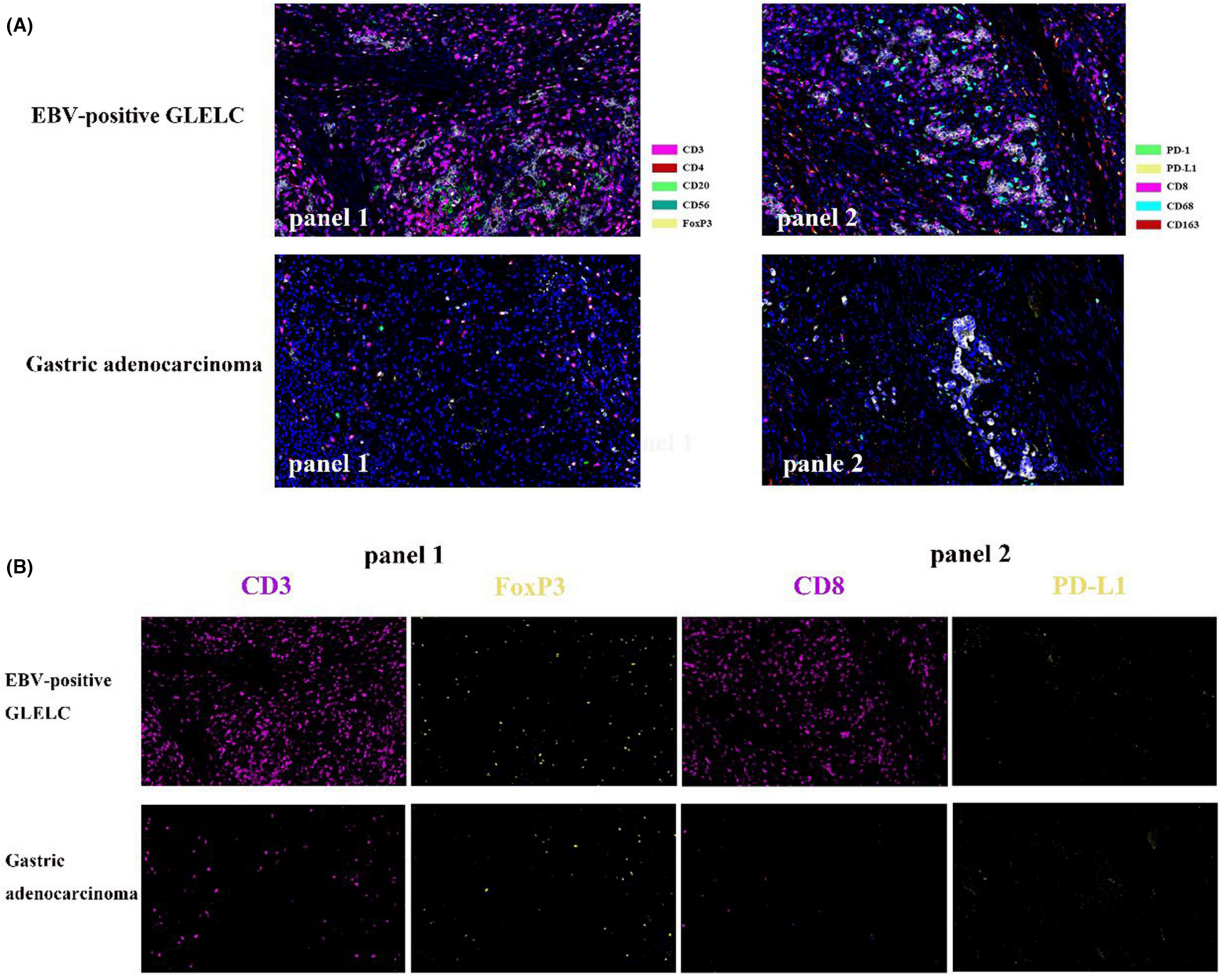
Multiplex staining of immune markers in patients with EBV‐positive GLELC and gastric adenocarcinoma. (A) Microphotographs of representative examples of multiplex IF markers in each group. (B) Illustration of the staining of CD3, CD8, Foxp3, and PD‐L1 in each group. ×200 magnification.

To confirm the TME heterogeneity of EBV‐associated GLELC, multiplexed immunofluorescence (mIF) staining was conducted for untreated tumor tissues. **Panel 1** and **Panel 2** were sequentially detected, and representative images were recorded as shown in Figures 2, 3.

**FIGURE 4:**
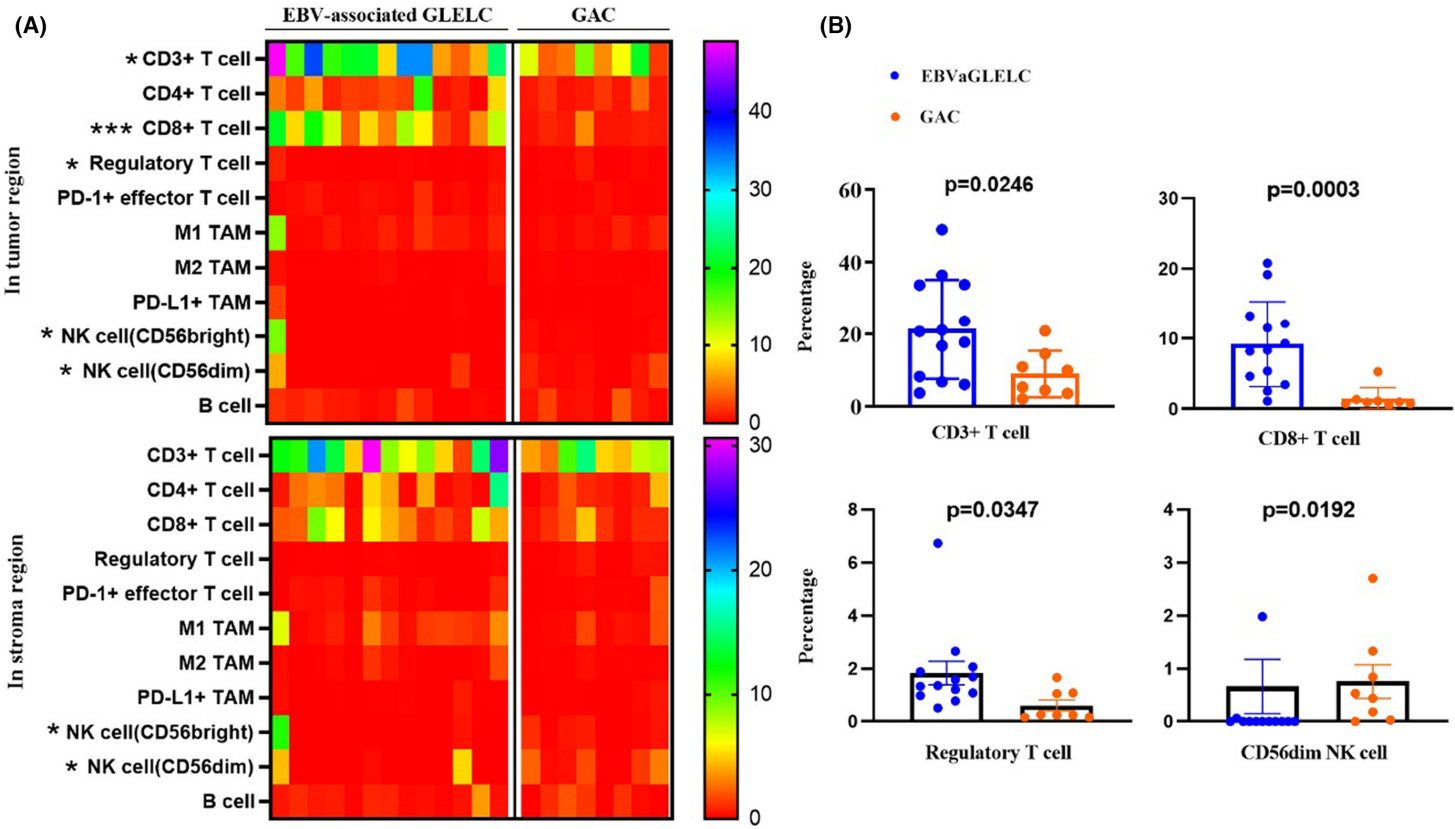
Evaluation of subgroups of immune cells using multiple immunofluorescence assays. (A) Immune cell profile for the patients with mIF staining test shown through a heatmap. (B) mIF analysis further revealed the distribution of CD3+ T cells, CD8+ T cells, Treg cells, and NK cells in the tumor region. Each dot represents one sample, and the data are presented as the mean ± SEM. *p*‐values are shown as *p < 0.05; **p < 0.01; ***p < 0.001; ****p < 0.0005.

Tumor classifications through characteristics of the tumor microenvironment have been proposed.^24^, ^25^ Based on the infiltration profile of different immune cells, tumors were grouped into hot and cold tumors. A comprehensive understanding of the cytotoxic T‐cell landscape within a tumor could better stratify patients and guide therapeutic strategies. Generally, tumors lacking or having few tumor‐infiltrating lymphocytes are considered immunologically “cold.”^26^ As shown in Figure 2, EBV‐positive GLELC displayed “hot” TME characteristics. In the tumor region, the frequencies of CD3^+^ T cells and CD8^+^ T cells were significantly elevated in EBVaGLELC compared with GAC (*p* = 0.0246 and *p* = 0.0003, respectively, Figure 4). Additionally, the percentage of Treg cells was significantly higher in EBV‐positive GLELC (*p* = 0.0347). The infiltration of NK cells was poor in EBV‐positive GLELC. Although Treg cells correlated with the formation of a cold TME in the tumor region and less NK cells infiltration in both the tumor region and stroma region, the proportion was far less than tumor‐infiltrating lymphocytes. Other markers did not show a significant difference.

**FIGURE 5:**
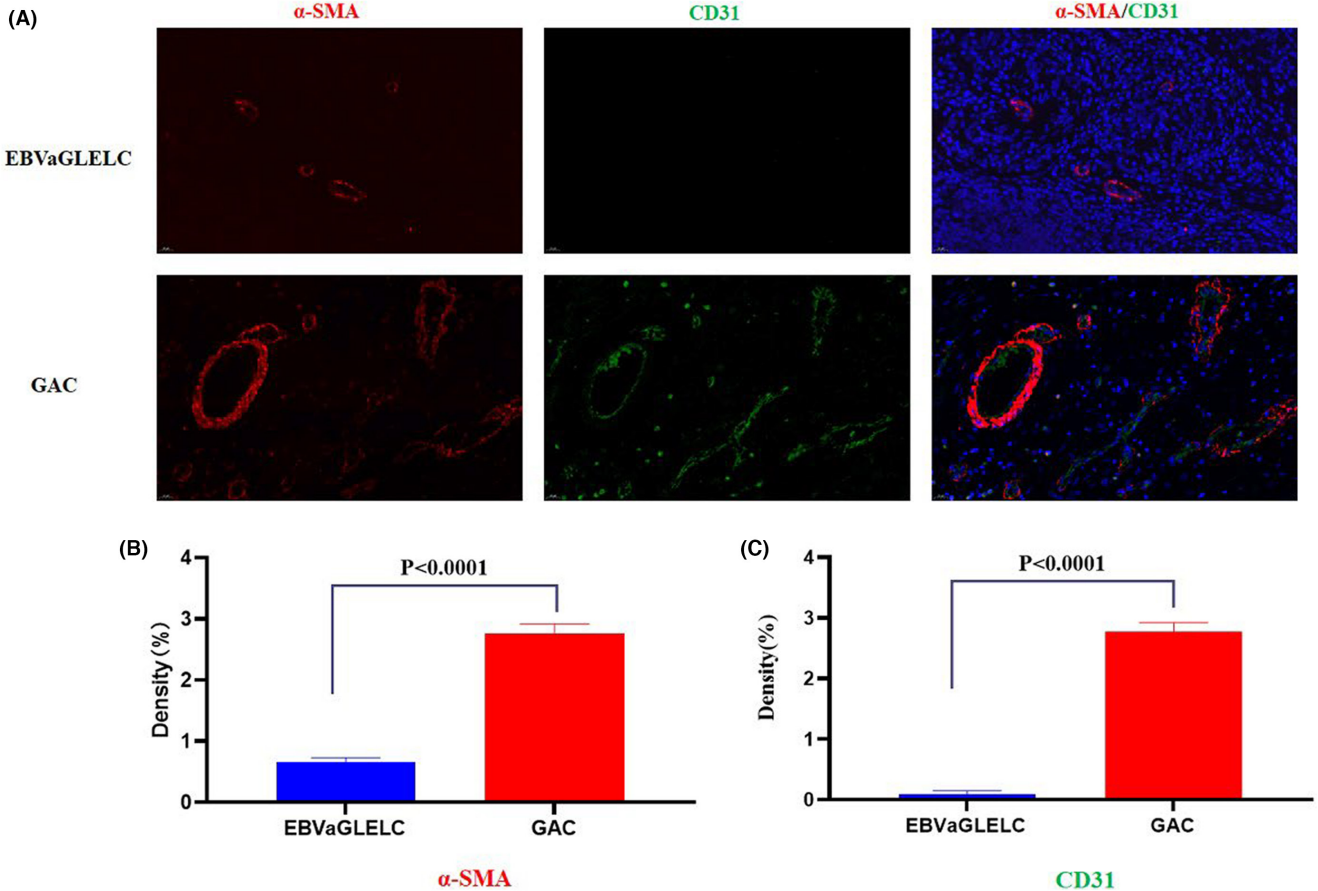
Evaluation of tumor vascular structure using multiple immunofluorescence. (A) The staining of α‐SMA and CD31 in patients. (B) Quantitative analysis of α‐SMA. (C) Quantitative analysis of CD31.

In addition, there were significant differences between tumor vascular structure assessed by CD31 and α‐SMA IF double staining (Figure 5A). The expression of α‐SMA protein in GAC was higher than that in EBV‐associated GLELC (*p* < 0.001, Figure 5B). The microvessel density, which was evaluated by CD31, in the GAC group was markedly greater than that in the EBV‐positive GLELC group (*p* < 0.001, Figure 5C).

### 3.4 A rare case of advanced GLELC

**FIGURE 6:**
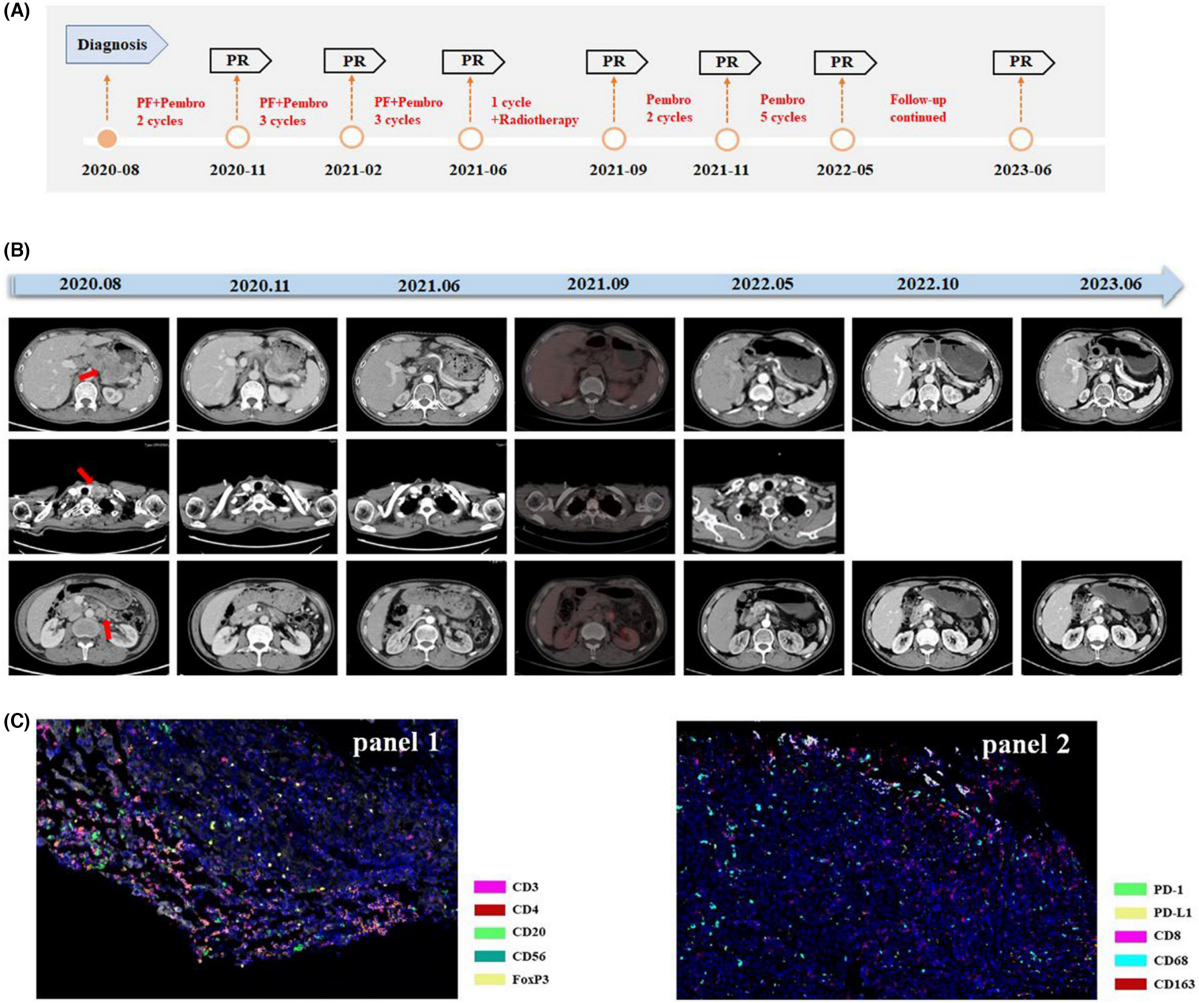
Treatment details, imaging radiographic findings, and mIF analysis of the case. (A). Timeline scheme of this patient. (B) Representative images of the major clinical event since diagnosis. (C) Immune cell distribution of this patient. Representative images for Panel 1. CD3, CD4, FOXP3, CD20, and CD56. Panel 2. CD8, PD‐1, PD‐L1, CD68, and CD163 expression in the TME. ×200 magnification.

A 52‐year‐old male was referred to the West China Hospital Sichuan (China) with a swallowing disorder in July 2019. Endoscopy analysis revealed an ulcerated lesion in the stomach. The biopsy was performed, indicating poorly differentiated carcinoma, and the subsequent immunohistochemistry results showed PCK (+), EMA (±), CDX2 (−), CgA (−), Syn (−), CD20 (−), CD3 (−), Ki67 (+, 50%), CK8/18 (+), P63 (+), CK5/6 (+), CK7 (−), and EBER1/2‐ISH (+) (Figure S1). Genetic testing showed PIK3CA mutations, and the CPS was 9. A computed tomography (CT) scan indicated that the lesion was a tumor with multiple lymphadenectasis and liver metastasis, and its transverse section measured 5.7 × 4.2 cm (Figure 6B **)**. After excluding the diagnosis of nasopharyngeal carcinoma, the patient was diagnosed with EBV‐associated GLELC and staged as IVA (T4a, N3b, cM1).

The patient was not considered for surgery. After obtaining consent for cancer treatment (pembrolizumab 200 mg d1, cisplatin 40 mg d1‐2, 30 mg day 3 and 5‐fluorouracil 1200 mg day1‐4, q3w), the patient commenced systemic treatment in September 2020. Sustained partial response (PR) was achieved based on CT or PET/CT evaluation after eight treatment cycles from September 2020 to May 2021. In June 2021, a biopsy through gastroscopy confirmed that the primary tumor had achieved a complete pathological response. Since the lymph node beside the left renal artery area did not decrease, radiotherapy was given in July 2021 (45 Gy/25 f). Then, the patient was changed to maintenance immunotherapy monotherapy. After seven cycles of maintenance therapy, this advanced patient still benefited. We performed mIF analysis to investigate the immune context before treatment (Figure 6C). Higher dense infiltration of CD3+ T cells (48.98%), as well as CD8+ T cells (20.82%), was found, and NK cells were enriched in the tumor region (CD56 bright 14.96%; CD56 dim 6.56%). In addition, the patient tolerated the whole treatment well.

## 4 DISCUSSION

EBV is an oncogenic virus that infects approximately 90% of the world's population.^27^, ^28^ It is closely associated with specific epithelial and lymphoid cancers, such as NPC and EBV‐associated GC.^29^ EBV not only contributes to the oncogenic process in malignant cancers but also serves as an important prognostic indicator, which has been shown to be associated with lower tumor stages and lower mortality rates.^30^ The role of EBV in the pathogenesis of NPC and its interaction with the TME have been increasingly well described.^31^ However, information about EBV‐associated GLELC has not been well studied, given its rarity.

EBVaGCs represent approximately 10% of all gastric cancers, and EBVaGC has distinct characteristics that distinguish it from EBV‐negative GC.^32^ A higher frequency in young people, more lymphoid stroma, fewer *Helicobacter pylori* (HP) infections, higher PD‐L1 expression, more PI3K/AKT pathway mutations, and better outcomes were reported in EBVaGC.^3^, ^8^, ^33^ Lymphoepithelioma‐like gastric carcinoma is a rare type of EBVaGC that was first reported in 1976.^34^ GLELC has special pathological characteristics, including stromal and intense lymphocytic infiltration.^35^ In addition to being associated with EBV infection, another pathological type of GLELC is microsatellite instability (MSI)‐high tumors.^36^ Min et al. revealed that EBV infection could serve as an independent predictor of survival in patients with LELC.^37^ The disease is usually prevalent among males, and compared with other subtypes of GC, patients with LELC had more upper locations, more indeterminate Lauren classifications, lower T stages, and lower lymphatic invasion.^38^ These features may suggest that this subtype of GC has a better survival rate than other subtypes of GC. However, information about this disease is still lacking, especially for treatment strategies in advanced patients.

Cancer immunotherapy has revolutionized cancer treatment both as monotherapy or in combination strategies, especially ICIs that block cytotoxic T lymphocyte‐associated protein‐4 (CTLA‐4) and the PD‐1/PD‐L1 axis.^39^, ^40^ Immunotherapy is mechanistically different from other treatment modalities that can target the TME and the tumor itself.^41^ However, the response rate to ICIs is still far from satisfactory. This may be correlated with the variability in the TME between different types of cancer.^42^ Thus, identifying biomarkers that affect efficacy is an urgent unmet need. The expression level of PD‐L1 is the most promising biomarker in current clinical practice, and patients with higher expression are more likely to respond to ICIs.^43^ Prior studies reported that patients with EBVaGC tend to show a higher PD‐L1 expression level.^44^ Nakano et al. revealed that PD‐L1 overexpression in EBVaGC was caused by the high focal amplification of CD274 or IFN‐γ‐mediated signaling via interferon regulatory factor 3 (IRF3).^45^ However, there was no significant difference in the levels of PD‐L1 expression between the two groups, which may be due to the limited number of patients in our study.

In addition, based on mIF, we thoroughly clarified the characterization of the TME in EBVaGLELC. The TME plays a key role in tumor formation and progression, providing a beneficial environment for tumor cell proliferation. With the notion of TME, a new tumor classification was also proposed, “hot” or “cold” tumors, which contribute to stratifying patients who may benefit from immunotherapy.^25^ Such classification of tumor immune phenotypes mostly focuses on T‐cell abundance.^46^ Effective antitumor immunity is mainly dependent on the orchestration of powerful T‐cell responses, and generally, CD3 and CD8 are identified as essential effectors of efficacy and representative of hot tumors.^47^ Abundant infiltration of CD3^+^ T cells and CD8^+^ T cells not only helps form a hot TME but also contributes to predicting ICB therapeutic efficacy.^48^, ^49^, ^50^ Research also shows the crucial role of CD4+ T cells in the antitumor immune response, and their interaction with dendritic cells (DCs) is significant for stimulating cytotoxic T lymphocyte function.^51^ Moreover, a recent study revealed that CD4^+^ T cells might reprogram the TME to a hotter TME by facilitating the normalization of tumor vessels.^52^ However, the infiltration of immunosuppressive cell subsets such as Treg cells, M2 TAMs, and myeloid‐derived suppressor cells (MDSCs) could impede antitumor T‐cell functions and lead to immunotherapy failure. Treg cells, characterized by the expression of Foxp3, have been proven to suppress the activation of CD8 cytotoxic T cells and CD4 helper T cells.^53^ TAMs can be classified into antitumor M1‐like or protumor M2‐like TAMs.^46^ M1 macrophages are associated with acute inflammation and antitumor activity, but M2‐like TAMs are correlated with protumorigenic chronic inflammation. In addition, B cells and NK cells also serve as components of the TME and can be linked to better clinical outcomes.^54^, ^55^

In our study, patients with EBVaGLELC exhibited an increased number of CD3+ T cells and effector T‐cell infiltration, suggesting that these patients may benefit from immunotherapy. Moreover, the fraction of Treg cells was enriched in EBVaGLELC tumors compared to GAC, which may be associated with an immunosuppressive TME and mediate resistance to ICIs. However, the proportion was far less than tumor‐infiltrating lymphocytes. Additionally, NK cells have the potential to magnify immune responses, and interest in harnessing NK cells for cancer immunotherapy is rapidly growing.^56^ However, fewer NK cells were found in the EBVaGLELC group than in the GAC group in this study. Other markers did not show a significant difference.

Additionally, vasculature networks in the TME are essential for tumor development and metastasis to other tissues.^57^ Vascular endothelial growth factor (VEGF) signaling is considered the main angiogenesis promotor.^58^ VEGF inhibits the function of immune effector cells, promotes the infiltration of immunosuppressive cells, and induces the expression of immune checkpoint molecules such as PD‐1.^59^ In this study, we observed that the tumor angiogenesis of EBVaGLELC was less intense than that of GAC, which may contribute to a favorable microenvironment.

Understanding the crosstalk between the TME and tumor cells is necessary in tumor immunotherapy to identify ICB responders, help predict prognosis, and guide immunotherapy. However, the complex connection in the TME has not been sufficiently illustrated until now. Here, we comprehensively studied the immune cells and the tumor vascular structure in EBVaGLELC, which may facilitate the understanding of the complex interaction between cancer cells and the TME and thus further encourage future research and guide treatment. Above all, EBV‐associated GLELC showed different patterns in immune cell infiltration and tumor vascular structure compared to patients with GAC. An immunostimulatory TME was shown in patients with EBV‐associated GLELC, which may raise the potential of checkpoint immunotherapy for this rare disease.

### 4.1 Limitations of the present study

There are some limitations in the present study. First, the number of patients in our study is limited due to a relatively low incidence of EBVaGLELC, and expanding the sample size is still needed in future research to verify our results. Second, we only assessed the TME before treatment due to the limited accessibility of tumor tissue during and after treatment. Dynamic changes in the tumor microenvironment could not be detected. Moreover, the results of analysis from biopsy and resection specimens may be discordant. Finally, single‐cell RNA sequencing or bulk RNA sequencing could provide more detailed information than the multiplexed immunofluorescence technique.

## 5 CONCLUSION

Compared with gastric adenocarcinoma, EBV‐positive GLELC has distinct TME characteristics. A “hot” TME may provide therapeutic advantages for those patients. Furthermore, our case provides an underlying therapeutic option for patients with EBV‐associated GLELC. Relevant clinical experiments are in preparation in our hospital to verify its clinical application value. Our study supplements the limited literature, which could also contribute to the clinic's treatment decision‐making, especially for advanced patients. Future studies with a larger sample size and a better design are still needed to verify our findings and enhance our understanding of EBV‐positive GLELC.

## AUTHOR CONTRIBUTIONS

**Yanna Lei:** Data curation (lead); investigation (equal); methodology (equal); validation (equal); writing – original draft (equal); writing – review and editing (equal). **Peng Cao:** Investigation (equal); visualization (equal); writing – original draft (equal). **Xiufeng Zheng:** Methodology (equal); supervision (equal). **Jing Wei:** Validation (equal). **Mo Cheng:** Validation (equal). **Ming Liu:** Conceptualization (equal); data curation (equal); supervision (equal); validation (equal).

## CONFLICT OF INTEREST STATEMENT

All authors declare no conflicts of interest concerning this work.

## ETHICS STATEMENT

Written informed consent was obtained from the patient for publication of this case report.

## Supporting information


**FIGURE S1.** EBER staining (200X) of tumor specimen which shows a number of EBER‐positive cells.

## Data Availability

All data relevant to the study are available from the corresponding author on reasonable request.
