# Supplementary figures and images for: Perspectives for immunotherapy of EBV‐associated GLELC: A relatively “hot” tumor microenvironment
Source: Cancer Med. 2023 Sep 21;12(19):19838–49. doi: 10.1002/cam4.6555 (PMC10587976; doi:10.1002/cam4.6555)

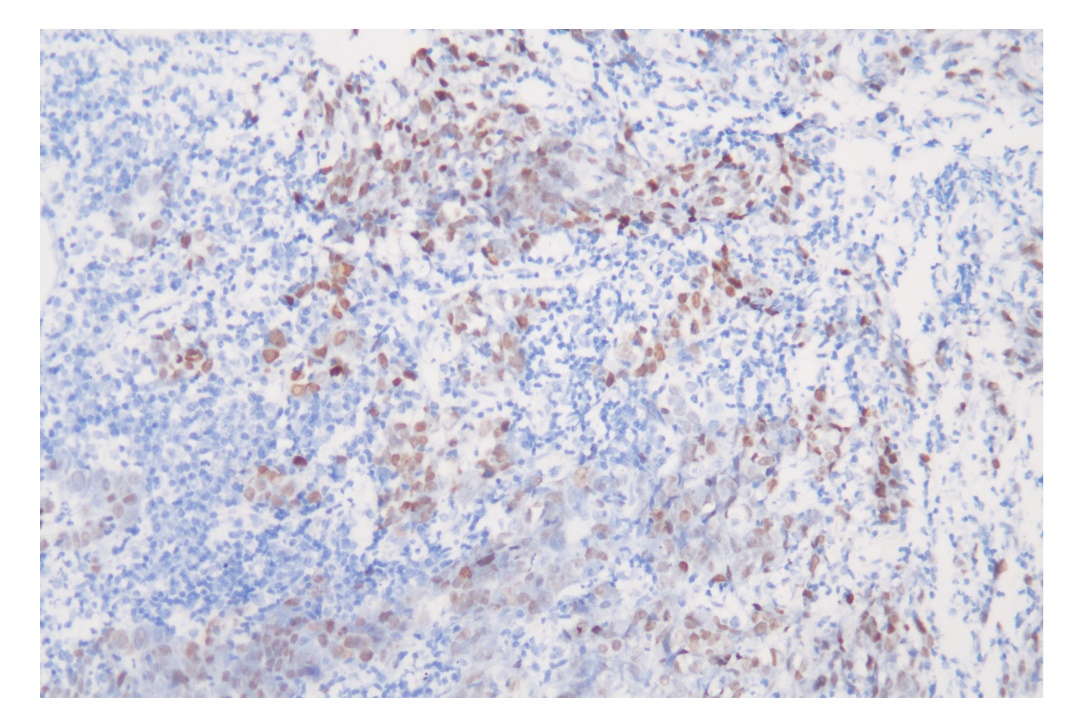


Fig. S1 EBER staining (200X) of tumor specimen which shows a number of EBER-positive cells.

Supplement: Supplementary file 1 — FIGURE S1. EBER staining (200X) of tumor specimen which shows a number of EBER‐positive cells. [file CAM4-12-19838-s001.docx]
